# A Case of Suicide by Dynamite

**Published:** 1881-12

**Authors:** 


### A CASE OF SUICIDE BY DYNAMITE.

This curious case of suicide is reported by Dr. Leadman in a late number of the *British Medical Journal* and, as he suggests, may prove of interest in a medico-legal aspect:

J. H., aged fifty-six, a well-sinker, of irregular and intemperate habits, on July 12th, concluded a "drinking-bout" of several weeks' duration. During this debauch, one evening, when in company with other men, a man of the party lost a purse containing seventeen pounds. A statement made by H. led to the apprehension and trial of a respectable farmer who was present when the purse was taken. The charge was proved to be groundless. On the 13th, the day of the trial, H., though sober and perfectly rational, failed to appear as a witness, making some excuses to his wife and son. About noon, at the time when he should have been in court, he walked into a yard at the back of his residence, and a neighbor in an adjoining field observing him suddenly fall went to his assistance. He saw blood issuing from his mouth, and at once sent for me. I found the mouth full of blood, and, on examination, the soft palate torn away, the fauces rent, the tongue detached and mutilated, the teeth broken off and splintered, the superior maxillary bones separated and extensively fractured—the fractures extending to the floors of the orbits. Blood was extravasated into the eyeballs, the lower eyelids, and the upper portions of the cheeks. The inferior maxillary bone was broken into about twenty pieces. The skin of the cheeks and lips was intact, save a few scratches on the internal surface of the latter. There was no charring of the tissues. A box of matches was found in his pocket, and one, partly consumed, close to his mouth where he fell. In his trade he used both cartridges and caps containing dynamite, and was well acquainted with this terrible explosive. One of these he had placed in his mouth, and, after igniting the short fuse attached to it, deliberately awaited the result. He survived the lesions two hours, remaining unconscious the whole time. The evidence given at the inquest was considered by the jury conclusive as regarded his insanity, and a verdict of *felo de se* was returned. Although I have both inquired of my friends and examined several works of reference I have failed to discover a similar case recorded.
—*Louisville Med. News.*

